# Disseminating Evidence-Based Interventions for Adolescent HIV Treatment and Prevention in Sub-Saharan Africa

**DOI:** 10.1007/s10461-022-03794-3

**Published:** 2022-09-27

**Authors:** Millicent Atujuna, Joseph Tucker, Natasha Crooks, Brian Zanoni, Geri R. Donenberg

**Affiliations:** 1 Desmond Tutu HIV Centre, Faculty of Health Sciences, School of Medicine, University of Cape Town, Level 1, Wernher Beit North Building, Cape Town, 7505 South Africa; 2 Division of Infectious Diseases, University of North Carolina at Chapel Hill, Chapel Hill, USA; 3 Department of Human Development and Nursing Science, University of Illinois at Chicago, Chicago, USA; 4 Departments of Medicine and Paediatric Infectious Diseases, Emory University School of Medicine, Atlanta, USA; 5 Department of Medicine, Center for Dissemination and Implementation Science (CDIS), University of Illinois at Chicago, Chicago, USA

**Keywords:** Dissemination, Evidence-based-interventions, Adolescent HIV, Sub-Saharan Africa

## Abstract

Over two-thirds of adolescents living with HIV worldwide reside in sub-Saharan Africa (SSA). Despite widespread availability and access to evidence-based HIV prevention and treatment, dissemination has been inadequate. This commentary distinguishes between implementation and dissemination, reflecting on the unique barriers to dissemination of evidence-based programs in SSA. We present a seven-strategy blueprint developed by United States Agency for International Development (USAID) that emphasizes targeted communication about research findings. Two case studies from the Adolescent HIV Implementation Science Alliance are presented to illustrate the value of planning for dissemination. We propose recommendations to strengthen dissemination recognizing that these may not be possible or appropriate in all situations, including developing a plan early in the process, engaging a dissemination technical team for support, the application of methodological rigor and theory to inform dissemination, active involvement of youth voices and digital tools to maximize message reach, and a keen recognition of evolving contexts and shifting priorities in order to nimbly tailor messages as needed.

## Background

Of the 1.75 million adolescents 10–19 years-old living with HIV worldwide, 1.5 million (88%) reside in sub-Saharan Africa (SSA) [1]. The disproportionate burden of HIV in SSA is further exacerbated by the uneven increase in new HIV infections in which adolescents make up more than half [2]. Compared to their male peers, adolescent girls and young women (AGYW) are especially vulnerable [3]. All told, AIDS remains the leading cause of death among adolescents in SSA [2, 4] despite the availability and free access to HIV prevention and treatment resources.

Evidence-based interventions (EBIs) targeting adolescent HIV prevention and treatment must be implemented at scale to achieve an AIDS-free generation, yet this requires effective dissemination practices. Many EBIs exist to address adolescent HIV prevention and treatment, including behavioural approaches (voluntary HIV counselling and testing, HIV treatment clubs for antiretroviral treatment (ART) adherence, skills building programs), biomedical strategies (management and treatment of sexually transmitted infections, ART for prevention, pre-exposure prophylaxis, post-exposure prophylaxis, medical male circumcision), and structural level interventions (financial incentives and economic empowerment) [5–7]. Each of these requires thoughtful attention to how best to inform and engage implementers to use these EBIs. Lack of attention to dissemination practices has, in part, led to poor awareness, low uptake, and minimal adoption of EBIs.

The terms “implementation” and “dissemination” are often used concurrently in discussions regarding the uptake and reach of EBIs. However, understanding the distinction between them is important to achieve more successful dissemination. Implementation science (IS) seeks to close the gap between what we know works and what is used in practice. IS seeks to identify the determinants that facilitate and/or hinder the delivery of EBIs in the real world and develop strategies to address them. Several theoretical frameworks exist to explain poor implementation outcomes and provide structure to evaluate strategies designed to combat barriers and enhance facilitators.

By contrast, dissemination is the intent to distribute information and spread knowledge about EBIs for public consumption. Dissemination is a critical but distinct component from implementation and required as a bridge to intervention scale up [8]. In the dissemination process, however, key components of what works and how they are identified using IS should be included in the dissemination. We argue therefore, that a symbiotic relationship exists between IS and dissemination. Wilson and colleagues suggest that dissemination is a planned process of sharing information about effective interventions and should include attention to the target audience, the settings in which research findings are received, and the manner of communication and interaction with wider audiences to facilitate uptake [9]. Inconsistent definitions and the use of different terms interchangeably, such as diffusion and knowledge translation, has challenged the creation of a systematic body of research on dissemination frameworks and theories [10]. Far less research has focused on dissemination strategies even as the literature supporting IS continues to grow.

Hence, in this commentary, we focus on dissemination specifically within the SSA context. We briefly review barriers to dissemination of EBIs in SSA and recommend a seven-strategy blueprint proposed by USAID to improve dissemination. We describe two case studies from the Adolescent HIV Implementation Science Alliance (AHISA) to illustrate the value of planning in advance for dissemination, and we close with key lessons learned through these projects.

### Barriers to Dissemination of Effective Programs

Many factors impede dissemination of EBIs. Barriers may be grouped into at least four domains—communication [9], researcher accountability, early planning, and resources. First, ineffective communication about EBIs, such as inadequate “publicity”, poor tailoring to the target audience, and unconvincing messaging all hinder dissemination. Messaging about how EBIs were developed, for whom they are best suited, why they are important, and their efficacy are essential to obtain buy-in and increase adoption. The most common ways academic researchers communicate about EBIs, namely through academic journals in the form of peer reviewed articles, systematic reviews, and meta-analyses, is insufficient to achieve diffusion of information or the reach required for greater uptake. These methods of dissemination are important to build a scientific knowledge base, but they fail to reach the relevant audiences and are unlikely to be read by front-line implementers [9–11].

Second, researchers frequently view dissemination as someone else’s responsibility—placing it in a separate silo from scientific discovery. But, researchers are arguably essential to dissemination efforts, since they know the most about the EBI, for whom it is appropriate, and its level of effectiveness. Collaborations between researchers and other key players who recognize the importance of dissemination (i.e., youth and implementers) can ensure that intervention designs carefully document the information needed for dissemination (target audience, content) and implementation (context, intervention materials used, desired outcomes), making it easier for future dissemination of EBIs [11–13].

Finally, poor dissemination results from an absence of advanced planning and insufficient resources to facilitate diffusion. For example, dissemination requires active “pushing out” of information about an EBI to potential implementers and policy makers, yet few studies build in personnel time and effort or resources to conduct these activities, in part because few funding opportunities allow dissemination efforts to be included in the budget. Likewise, few implementing agencies build in time and effort for their staff to seek out new innovations, particularly when resources are depleted or barely available to complete the agency’s mission [14]. Addressing these and other barriers to dissemination is critical to improve implementation outcomes, such as reach, adoption, acceptability, and sustainability, as well as clinical outcomes.

For dissemination to achieve a public health impact, the strategies and frameworks must integrate and utilize evidence-based mechanisms associated with persuasive communication [15], diffusion of innovation [16], and social marketing theories [17]. Wilson and colleagues recommend McGuire’s five elements that align with persuasive communication theory to guide dissemination efforts. Specifically, dissemination must consider the (a) source of the communication, (b) communication channels, (c) message itself, (d) audience, and (e) the setting or context where the EBIs will be implemented [9]. Consistent with these elements, *active dissemination* [9] targets key audiences that are directly involved in the implementation of EBIs and the individuals tasked with deciding what EBIs to adopt. Finally, effective dissemination depends on recognizing that dissemination occurs in phases along a continuum from increasing knowledge of EBIs, to persuasion, implementation, and finally confirmation [9] (Table 1)

**Table 1:** Terms and definitions

Definition of key terms included in the article content	
Dissemination	A planned process that considers the target audience, the setting in which research findings are be to be received, and the manner of communicating and interacting with the wider audiences to facilitate the uptake and understanding of research [1] or A targeted distribution of information and intervention materials to a specific public health or clinical practice audience [2]
Implementation	The use of strategies to adopt and integrate evidence-based health interventions and change practice patterns within specific settings
Implementation science	The systematic study of how a specific set of activities and designed strategies are used to successfully integrate an evidence-based public health intervention within specific settings (e.g., primary care clinic, community centre, school) [3]
Diffusion	A process of that occurs as people adopt a new idea, product, practice or philosophy [4]
Knowledge translation	A process in which an evidence based public health intervention is successfully integrated into established practice and policy [5]
Description of key theories employed in dissemination frameworks as described in Wilson et al. 2010	
Persuasive communication	Any message that is intended to shape reinforce or change the response of another or others [6]
Diffusion of innovation	A process that occurs as people adopt a new idea, product, practice or philosophy [4]
Social marketing	A process that applies marketing principles and techniques to create, communicate, and deliver value in order to influence behavior that benefits society (public health, safety, the environment and communities) as well as the target audience [7]
References	1. Wilson PM, Petticrew M, Calnan MW, Nazareth I. Disseminating research findings: what should researchers do? A systematic scoping review of conceptual frameworks. Implement Sci. 2010;5:91 2. Schillinger D. An introduction to effectiveness, dissemination and implementation research. UCSF Clinical and Translational Science Institute (CTSI) Resource Manuals and Guides to Community-Engaged Research San Francisco: University of California. 2010 3. Bauer MS, Damschroder L, Hagedorn H, Smith J, Kilbourne AM. An introduction to implementation science for the non-specialist. BMC Psychol. 2015;3(1):32 4. Kaminski J. Diffusion of Innovation Theory. Canadian journal of Nursing Informatics. 2021;16(3–4) 5. Chapman E, Haby MM, Toma TS, de Bortoli MC, Illanes E, Oliveros MJ, et al. Knowledge translation strategies for dissemination with a focus on healthcare recipients: an overview of systematic reviews. Implement Sci. 2020;15(1):14 6. McGuire W. Input and Output Variables Currently Promising for Constructing Persuasive Communications. 2001. In: Public communication campaigns [Internet]. SAGE Publications,: Thousand Oaks, CaliforniaInc. 3. [22–48] 7. Quinn GP, Ellery J, Thomas KB, Marshall R. Developing a Common Language for Using Social Marketing: An Analysis of Public Health Literature. Health Marketing Quarterly. 2010;27(4):334–53

The United States Agency for International Development’s (USAID) Research Technical Assistance Center (RTAC)^1^ [18] developed a blueprint for dissemination that combines persuasive communication strategies with design elements and audience tailoring. The blueprint lists seven components which culminate in accessible and visually appealing communication products to improve dissemination. The components can be tailored for use by policy makers, funders, implementers, and other interested groups [18].

**Fig. 1:**
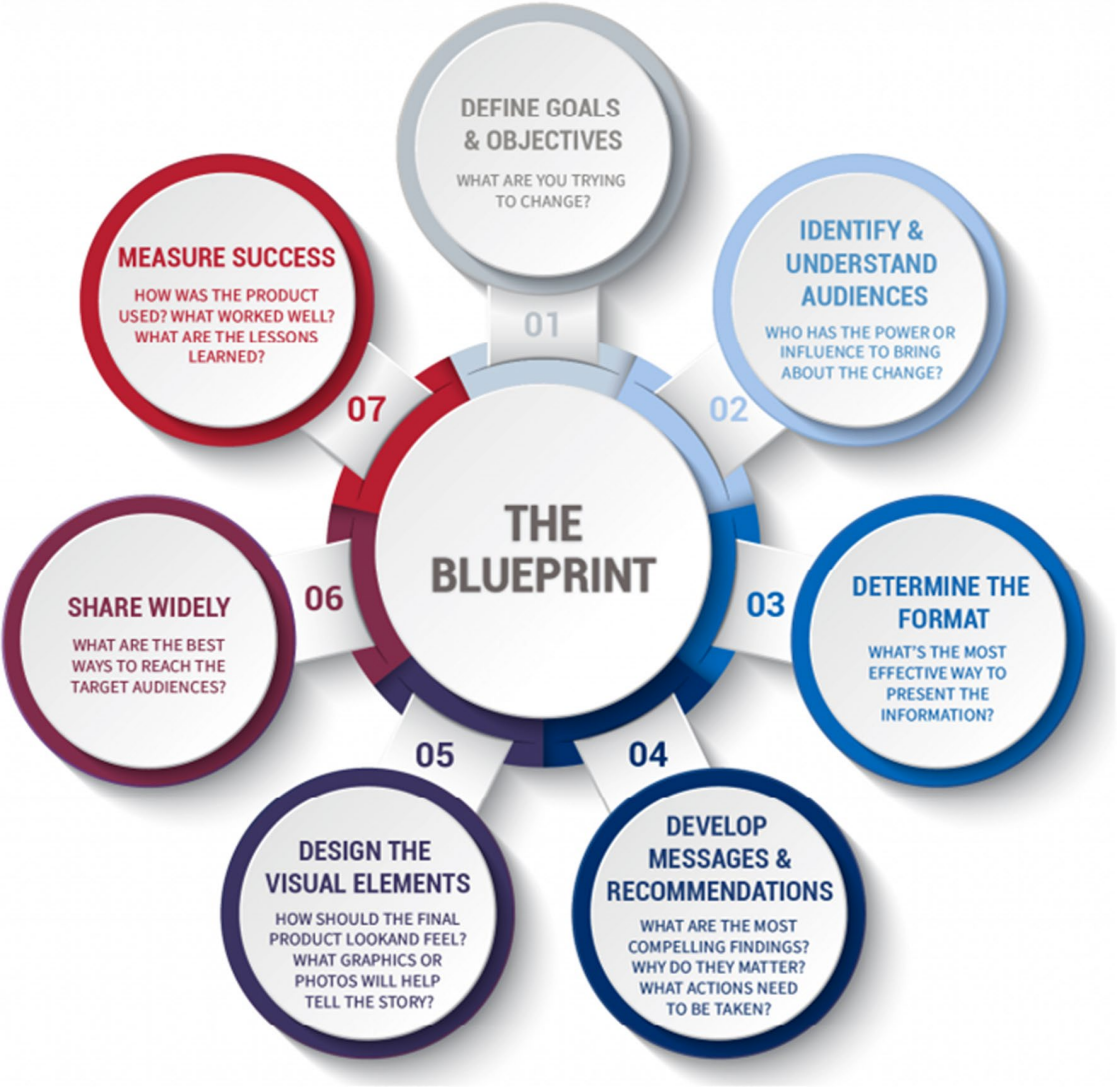
Seven strategies for research translation: a framework for more effective research communication and dissemination. Adapted from USAID, Research Technical Assistance Center: Research to Action Model, available at Available from: https://www.rtachesn.org/stories-and-news/a-blueprint-for-strategically-communicating-research-for-development-impact

Figure 1 illustrates the seven components hypothesized to guide successful dissemination of an EBI. One of the elements of the RTAC model is the importance of establishing a “dissemination technical team” to support the researchers seeking to develop a dissemination plan during the early phases of a project. The technical team may include individuals with expertise outside the content of the EBI but who bring unique expertise in communication and policy development. The technical team should understand the audience and the scope of the dissemination, and in this way, complement the researchers’ expertise. The technical team and researchers can work together to identify and define aspects central to dissemination and set dissemination goals and objectives. For example, dissemination goals might include ensuring easy access to the EBI or creating awareness and improving attitudes towards the EBI to encourage use and uptake. Likewise, the technical team can help identify and engage/involve target audiences, stakeholders, and implementers at different stages of the research process. Technical team members with direct connections to policy makers may also facilitate improved dissemination within health ministries. They can help the researchers identify the best channels for dissemination, platforms for wider viewership, and elements of the product that should be highlighted for maximum appeal. Finally, the technical team can also ensure the implementation of monitoring and evaluation tools to track and measure successful dissemination or areas in need of improvement.

Another strategy to improve uptake of EBIs is developing strategic dissemination plans that will facilitate broader awareness of effective programs. One example where dissemination was highly effective is the Kenyan uptake of pre-exposure prophylaxis (PrEP), which has surpassed most other African countries. In May 2017, there was zero uptake of PrEP in Kenya. Since the *Jipende JiPrEP* (meaning “Love yourself, PrEP yourself”) campaign was introduced, over 25,000 at-risk individuals have initiated PrEP [19–22]. To promote PrEP dissemination, the Kenyan government developed a framework to implement PrEP using clear communication and an advocacy plan. The objective of the dissemination plan was to strengthen knowledge about PrEP services and access points, improve perceptions and attitudes toward PrEP among stakeholders, and increase demand for PrEP among the target audience [22]. The plan detailed key communication channels and positioning statements that were achievable. They also pursued structured and systematic engagements with community stakeholders to dispel misconceptions about PrEP. Of course, other strategies were put into place along with more effective messaging and communication that contributed to the success of the PrEP rollout. Still, this is an excellent example of how a dissemination plan can improve uptake of an EBI and achieve significant public health benefits. This example also highlights the important role stakeholders (Kenyan government in this case) play in championing and advocating for the need to improve the uptake of EBIs. Zambia implemented a similar PrEP implementation framework in April 2017, and by 2019 over 16,000 individuals had initiated PrEP [23].

## Case Studies from the Adolescent HIV Implementation Science Alliance (AHISA)

In 2018, the National Institute of Child Health and Human Development with support from National Institute on Minority Health and Health Disparities (NIMHD) and the Office of Behavioral and Social Sciences Research (OBSSR) established the HIV Prevention and Treatment through a Comprehensive Care Continuum (PATC^3^H) Consortium to reduce HIV incidence and improve HIV care among adolescents and young adults (AYA) in low and middle-income countries [24]. PATC^3^H includes eight research studies using a combination of interventions aimed at multiple levels (individual, interpersonal, community, structural) to improve adolescent health outcomes and reduce the 17-year gap between establishing the efficacy of an EBI through research and actually delivering the EBI in real-life settings [25]. As part of the application for funding, PATC^3^H required a model for dissemination that engaged stakeholders and decision makers in-country at the start of the research process to ensure uptake of effective EBIs. Subsequently, PATC^3^H joined forces with the AHISA [26] to facilitate IS efforts within the consortium. The research studies that comprise PATC^3^H and AHISA initiated in-country stakeholder engagement at the start of their projects to determine how best to address adolescents’ HIV-related health challenges. These kinds of engagements sought to bridge the gap between research and practice and serve as connective tissue—to explicitly facilitate the flow of relevant information and influence between all the concerned parties or stakeholders.

In the remainder of this commentary, we present two case studies from the AHISA/PATC^3^H collaboration to illustrate the application of USAID’s dissemination framework [27]: 4YouthByYouth (4YBY) [28], part of Innovative Tools to Expand Youth-Friendly HIV Self-Testing, and Informed, Motivated, Aware and Responsible Adults and Adolescents (IMARA-South Africa).

### Case Study 1

4YBY used a youth participatory action research framework to enhance youth engagement in HIV self-testing and related HIV prevention activities in Nigeria [28]. The project used crowdsourcing open calls and youth training to build capacity for youth engagement. Crowdsourcing is a technique where a group of individuals solve a problem and then share solutions with the public [29]. 4YBY used five of the seven strategies in the framework as part of its dissemination plan. First, consistent with Strategy 1 of the blueprint, they established a broad*objective* to increase HIV-testing among AYA in SSA, especially Nigeria. They used crowdsourcing and related participatory activities to increase HIV self-testing uptake among AYA. Next, consistent with Strategy 3, they created *platforms/formats* using both digital (Unstructured Supplementary Service Data (USSD), website, social media) and in-person approaches via AYA networks. USSD is a communications protocol that sends short text messages and does not require a smart phone. The project used USSD to optimize dissemination of HIV self-testing among youth with poor bandwidth or limited internet access (see Strategy 6). As recommended by USAID Strategy 2, the 4YBY website provided detailed information *uniquely* tailored to the AYA and included ongoing activities, including AYA blog posts, archived webinars, and details about participatory activities. The 4YBY social media platforms also offered a mechanism to livestream events about research findings, connect with interested AYA, and mobilize community interest. These activities are in line with Strategy 5 of the blueprint. The 4YBY team also disseminated study findings through in-person events. For example, the annual Nigerian Institute of Medical Research symposium offered *a formal way to share* research findings with the Lagos, Nigeria research community consistent with Strategy 6.

Drawing on USAID Strategy 4, the 4YBY team created several *infographics/visuals* as part of a co-creation process [30]. Infographics are visual displays of information that can be easily understood by a public audience, expand the potential audience, and enhance dissemination efforts. Infographics tailored for policymakers may be particularly useful to spur change. AYA within the 4YBY group worked in partnership with other AYA to co-create the infographics that resonated with Nigerian AYA and were relevant to the local context. The 4YBY team also developed videos to accompany peer-reviewed research manuscripts to communicate results, consistent with Strategy 5. For example, four AYA created a three-minute video indicating the *key messages* related to a scoping review on AYA engagement in HIV research studies. The video was designed, edited, and delivered by AYA who responded to the crowdsourcing open calls or related participatory events. The images and videos created by the 4YBY team have been used by the World Health Organization to enhance dissemination of research among AYA in sub-Saharan Africa.

### Case Study 2

IMARA-South Africa is evaluating a comprehensive HIV prevention package that includes an evidence-based mother-daughter behavioral intervention (IMARA) [27] to reduce incident HIV and STI infections, increase HIV-testing and PrEP uptake, and reduce risky sexual activity among South African AGYW. IMARA was initially developed and tested in the United States with 14–18 year-old African American girls and their female caregivers and demonstrated reductions in STI incidence at 12-month follow-up [31]. In South Africa, the study proceeded in two phases with slightly different dissemination goals. In phase one, the main *objective* was to adapt the curriculum to the South African context and engage stakeholders to increase enthusiasm and buy-in, consistent with USAID Strategy 1 of the blueprint. In phase two of the study, the main *objective* was to increase uptake of the IMARA-South Africa, improve future implementation, and promote rollout to other health clinics.

More specifically, IMARA-South Africa illustrates the close relationship between IS and dissemination described earlier [32, 33]. Two of the strategies recommended in the blueprint were central features of IMARA-South Africa, namely engaging relevant *audiences early in the research process* (Strategy 2) and tailoring the *aspects/format of the intervention* (Strategy 3) to fit the local context. Grounded in IS, the researchers explored the needs of the community and the context/setting for the IMARA-South Africa to understand components that would benefit the target audience. They engaged community stakeholders, community advisory boards, mothers, and AGYW to guide changes to the intervention curriculum and obtain feedback on how to implement a mother-daughter sexual health research project [34]. Consistent with USAID Strategy 5, the project intentionally sought feedback from different stakeholders to *identify the unique messages for each audience* and understand how IMARA-South African should be disseminated. Each component of the intervention was vetted with the target population, and individuals offered feedback pertaining to its feasibility, appropriateness, sustainability, and key messages. Finally, the IMARA-South Africa team created a collaborative system to regularly share findings with *program implementers* (Strategy 6) in key country-level health departments, including the Department of Health (DOH). The team’s goal was to stay current on local priorities for sexual health education of AGYW, understand relevant country-wide policies, and consider how best to implement IMARA-South Africa in the future. Initial feedback helped the research team align study activities and content with the vision of the DOH and equip communities with skills to prevent infections including HIV (Table 2).

**Table 2:** Relationship between the USAID recommended strategies and strategies implemented in the two case studies

USAID recommended strategies	Strategies implemented in the 4YBY case study	Strategies implemented in the IMARA-South Africa case study
Strategy 1: Define goals and objectives – *What are you trying to change?*	– Established a broad objective* to increase HIV-testing among AYA in SSA, especially Nigeria – Employed crowdsourcing and related participatory activities to increase HIV self-testing uptake among AYA	– Sought to increase uptake of the IMARA-South Africa intervention, improve future implementation, and promote rollout to other health clinics
Strategy 2: Identify and understand audiences – *Who has the power or influence to bring about the change?*	– The 4YBY website provided detailed information ***uniquely tailored to the AYA*** and included ongoing activities, including AYA blog posts, archived webinars, and details about participatory activities – Social media platforms also offered a mechanism to livestream events about research findings, connect with interested AYA, and mobilize community interest	– Engaged relevant ***audiences early on in the research process including*** exploring the needs of the community and the context/setting for the IMARA-South Africa to understand components that would benefit the target audience
Strategy 3: Determine the format – *What is the most effective way to present the information?*	– Created ***platforms/formats*** using both digital (Unstructured Supplementary Service Data (USSD), website, social media) and in-person approaches via AYA networks	– Tailored the ***aspects/format of the intervention*** to fit the local context
Strategy 4: Develop messages and recommendations – *What are the most compelling findings?* – *Why do they matter?* – *What actions need to be taken?*	– Created several ***infographics/visuals*** as part of a co-creation process	N/A
Strategy 5: Design the visual elements – *How should the final product look and feel?* – *What graphics or photos will help to tell the story?*	-Social media platforms also offered a mechanism to livestream events about research findings, connect with interested AYA, and mobilize community interest	– Sought feedback from different stakeholders to ***identify the unique messages for each audience*** and understand how IMARA-South Africa should be disseminated – Vetted each component of the intervention with the target population – Sought information pertaining to intervention feasibility, appropriateness, sustainability, and key messages
Strategy 6: Share widely – *What are the best ways to reach the target audiences?*	Used annual Nigerian Institute of Medical Research symposium used to share information widely	– Created a collaborative system to regularly share findings with ***program implementers*** in key country-level health departments, including the Department of Health (DOH)
Strategy 7: Measure success – *How was the product used?* – *What worked well?* – *What are the lessons learned?*	N/A	N/A

Bold italic represents strategies implemented in both IMARA-SA and 4YBY case studies that align with the USAID recommended dissemination strategies

### Lessons Learned and Future Recommendations

Dissemination of evidence-based interventions is essential to maximize the impact of innovations, eliminate health disparities, and ensure health equity. The two examples offer lessons for researchers, practitioners, and policy makers seeking to improve dissemination of EBIs moving forward. First, the use of digital tools can enhance dissemination of evidence-based interventions among adolescents. Digital tools can be leveraged to promote sharing key messages, encourage discussions in real time, and obtain end-user feedback. However, care must be taken to recognize the digital divide between those with and without internet access, and dissemination strategies must consider how to reach offline audiences, for example, where target audiences congregate (e.g., after-school programs, clubs). Second, the case examples demonstrate how youth can play a key role in dissemination efforts by including their voices, expertise, and wisdom. They can help tailor dissemination materials and enhance their impact. Including youth also builds capacity for subsequent adolescent-led programs and research and creates a pipeline of future leaders. Third, effective dissemination must consider that contexts evolve and priorities shift. Both studies experienced challenges as a result of the COVID-19 pandemic. This required flexibility and adaptability as teams were forced to adopt new dissemination methods to convene stakeholder meetings and in some cases move research activities into digital spaces. Fourth, both studies illustrate the importance of teamwork and collaboration to achieve their objectives. Dissemination requires partnerships between implementers and researchers in order share findings and target messages to the relevant audiences. Fifth, it is worth noting that neither case study initially budgeted for dissemination activities. However, as suggested above, with early and careful planning, they were able to implement strategies that facilitated dissemination. Finally, there is a critical need for more rigorous measurement and evaluation of dissemination strategies in order to optimize implementation and bridge the know-do gap.

## Conclusion

Dissemination is necessary to realize the full public health impact of evidence-based interventions and ensure all populations benefit equally from scientific advances. USAID offers a blueprint on how to plan for and carry out dissemination, and allows for more methodological rigor to inform the science of dissemination. Still, because dissemination is seldom incentivized in funding calls, it is rarely included in budgets making it difficult to implement. Future funding opportunities should explicitly require plans for dissemination that include budgetary considerations. Even without this, however, investigators should consider and plan for dissemination in their study timeline and design.

## Footnotes

1 Research Technical Assistance Centre (RTAC) is a net of researchers ‘supporting USAID’S mission bureaus, and independent offices to make evidence-based decisions, serving as a strategic resource to USAID. RTAC leverages the scientific expertise of the academic researchers to provide timely technical assistance’. https://www.rtachesn.org/. Based on the RTAC model, and given the need to have evidence-based interventions disseminated for impact, we suggest research projects establish and build within their project teams a dissemination team to move the dissemination agenda, taking components of the interventions that need to be disseminated from the beginning to the end of the study.

## Declarations

### Research Involving Human and Animals Rights

The case studies presented in this commentary received ethics approvals. IMARA was approved by the Health Sciences Ethics committee (HREC) at the University of Cape Town, South Africa with reference number (077/2019). The 4YBY study received ethics approval from the Nigerian Institute of Medical Research (IRB/18/028) and St. Louis University (30347).
